# Cutaneous metastasis of signet ring cell carcinoma

**DOI:** 10.1016/j.jdcr.2025.11.044

**Published:** 2025-12-11

**Authors:** Dakarai Dunbar, Silanur Inanoglu, Thomas Whiting, Mark Lewis, Madison Hill, Hannah Kim, Jean Margaret Moresi, Aisha Mumtaz

**Affiliations:** a Department of Dermatology, University of Maryland School of Medicine, Baltimore, Maryland; b Department of Pathology, University of Maryland School of Medicine, Baltimore, Maryland

**Keywords:** cutaneous malignancy, cutaneous metastasis, gastrointestinal malignancy, pathology, signet ring cell carcinoma, skin biopsies

## Introduction

Signet ring cell carcinoma (SRCC) is a variant of adenocarcinoma predominantly characterized histologically by eccentric, irregular nuclei and abundant intracytoplasmic mucin, resembling a “signet ring.”^1^ SRCCs most frequently arise in the stomach; however, they may also originate elsewhere in the body, such as in the breast, colon, esophagus, rectum, and lungs.^2^^,^^3^ Although cutaneous metastases of gastric SRCCs to the skin are rare, reported presentations have been highly variable, ranging from firm nodules to lesions resembling allergic contact dermatitis on the face, to scar-like indurated lesions in the epigastric region, and to erysipelas-like lesions on the head and neck.^4^, ^5^, ^6^ We report a 47-year-old female patient who presented with a 2-month erythematous plaque on the abdomen. The biopsy of the lesion revealed metastatic gastrointestinal signet ring cell carcinoma, which was diagnosed expeditiously due to its cutaneous manifestation.

## Case report

**Fig 1:**
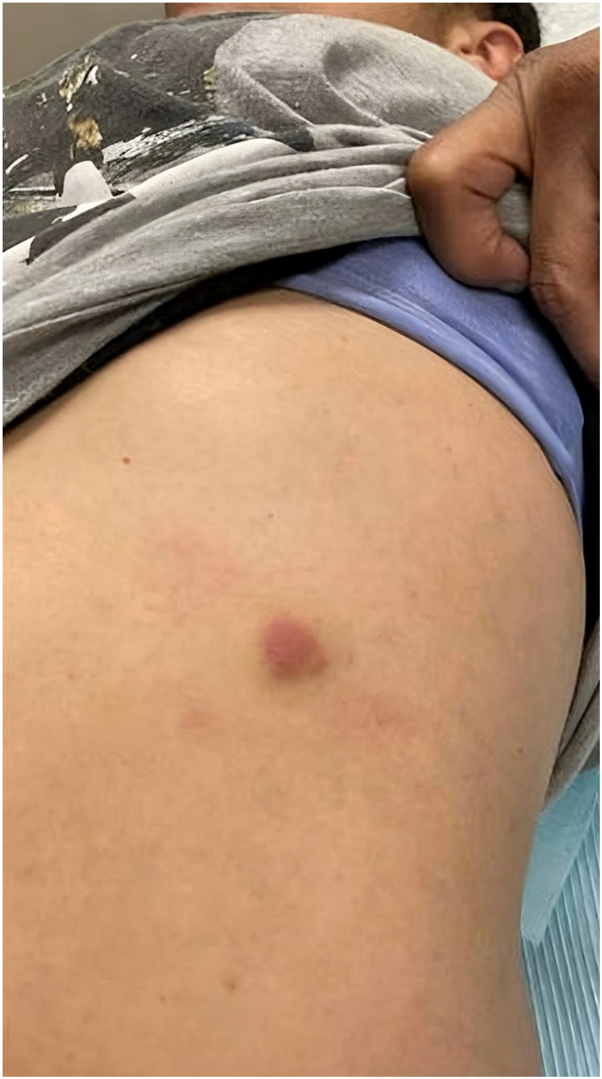
Solitary erythematous nodule on the left abdomen.

A 47-year-old woman presented to the dermatology clinic with an erythematous nodule on the left of her abdomen. The lesion was asymptomatic but slowly growing over the last 2 months, which prompted her evaluation, as seen in Fig 1. Her medical history was notable for primary hypertension, hyperlipidemia, alcohol use disorder, and hepatic steatosis. She did not endorse any systemic symptoms at the time of visit. There was no personal history of malignancy. Medications included clonidine, atorvastatin, and naltrexone, with no recent changes. She did not endorse any systemic symptoms at the time of the visit.

**Fig 2:**
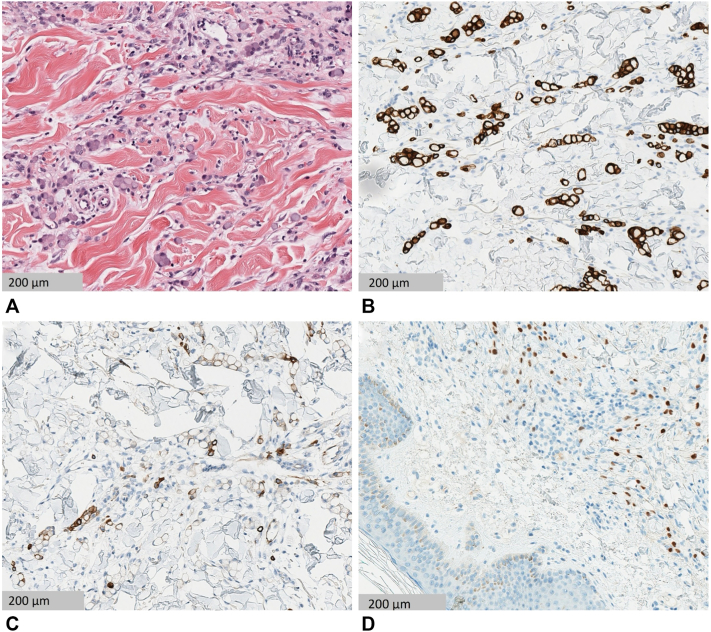
Hematoxylin-eosin staining shows single and aggregates of signet ring cells seen in the reticular dermis. There are central globoid cytoplasmic mucin droplets with eccentrically placed nuclei in the neoplastic cells **(A)**. CK 20 and CK 7 **(B** and **C,** respectively**)** show cytoplasmic staining in the tumor cells, and CDX-2 **(D)** shows nuclear staining in the tumor cells.

The punch biopsy showed aggregates of cells with central globoid cytoplasmic mucin droplets and eccentrically placed nuclei in a “signet ring” appearance throughout the reticular dermis. Aggregates of signet ring cells around peripheral nerves were also noted. There was a surrounding palisaded lymphohistiocytic infiltrate. The atypical cells were highlighted by immunoperoxidase stains for cytokeratin (CK) 20 and 7 and caudal type homeobox (CDX) 2, as seen in Fig 2. The cells were also positive for cytokeratin antibody mixture 5.2 (CAM5.2) and faintly positive for special AT-rich sequence-binding protein 2 (SATB2), while staining for GATA binding protein 3 (GATA3) and thyroid transcription factor-1 was negative. Next-generation sequencing of the biopsy tissue showed cells that were claudin-18 (CLDN18) positive and human epidermal growth factor receptor 2 (HER2) negative, with a CLDN18::ARHGAP26 (rho GTPase-activating protein) pathogenic fusion. Immunohistochemistry testing for mismatch repair proteins indicated a low probability of microsatellite instability. Given the immunophenotypic profile, the findings favored a gastrointestinal tumor, consistent with metastatic signet ring cell carcinoma. The patient was promptly referred for oncologic care.

Positron emission tomography (PET) and computed tomography (CT) later revealed multiple metabolically active osseous lesions involving both the axial and appendicular skeleton, consistent with metastatic disease. Initial PET also showed increased uptake in the gastric wall; however, esophagogastroduodenoscopy (EGD) and colonoscopy failed to demonstrate any malignant lesions that correlated with PET scan activity.

The patient was unable to schedule a colonoscopy and endoscopy for multiple months after initial biopsy, so given the high suspicion for systemic malignancy, empiric chemotherapy with FOLFOX therapy (folinic acid, fluorouracil, and oxaliplatin) was initiated prior to completion of endoscopic evaluation. Zolbetuximab was subsequently added after CLDN18.2 positivity was confirmed on tumor immunohistochemistry. The patient is continued on systemic therapy today and continues to demonstrate an ongoing clinical and radiographic response. At the most recent follow-up, there was no evidence of recurrent or progressive malignancy.

## Discussion

Cutaneous metastases occur in approximately 0.7% to 9% of patients with internal malignancies and may be the first sign of an otherwise occult cancer.^2^ In our patient, the lesion’s subtle, nonspecific clinical morphology—a solitary, erythematous plaque overlying a firm subcutaneous nodule—underscored the importance of maintaining a broad differential and proceeding promptly with biopsy in cases of unexplained skin changes. Histopathology revealed signet ring cells containing intracytoplasmic mucin, a feature that is highly suggestive of adenocarcinoma. The immunoprofile—positivity for CK7, CK20, and CDX2—was suggestive of a gastric origin, though this pattern can also occur in colorectal signet ring cell carcinoma.^7^ However, the CLDN18-ARHGAP fusion is a molecular characteristic and independent prognostic indicator of gastric tumors, confirming that this patient’s SRCC likely originated in the stomach.^8^ Given the absence of overt gastrointestinal symptoms and an unremarkable family history for gastrointestinal malignancy, systemic staging was essential to confirm the primary site and assess disease burden. PET/CT, EGD, and colonoscopy were ordered to localize the tumor and identify additional metastatic foci. However, logistical barriers delayed these studies, posing a challenge in initiating timely treatment. In the setting of metastatic adenocarcinoma and concern for disease progression, empiric chemotherapy was initiated with the FOLFOX regimen, a standard first-line therapy for advanced gastrointestinal adenocarcinomas.^9^ FOLFOX was selected for its proven efficacy in both gastric and colorectal cancers and its relatively favorable toxicity profile in patients with preserved functional status (Eastern Cooperative Oncology Group 0). Zolbetuximab, a monoclonal antibody targeting claudin-18.2, was subsequently added after CLDN18.2 positivity was confirmed, which has demonstrated survival benefit in patients with advanced gastric or gastroesophageal junction adenocarcinoma expressing the target antigen.^10^ Although subsequent EGD and colonoscopy failed to identify a visible primary lesion, PET/CT findings of diffuse osseous metastases and mild gastric wall fluorodeoxyglucose uptake remained most compatible with an advanced gastrointestinal malignancy of primary gastric origin.

This case illustrates the potential for cutaneous metastasis to precede the detection of visceral malignancy, the critical role of histopathology and immunohistochemistry in narrowing the diagnostic possibilities, the impact of social and logistical factors on staging timelines, and the rationale for empiric initiation of a standard chemotherapy regimen when performance status is preserved and disease burden is high.

## Conflicts of interest

None disclosed.

## References

[1] Efared B., Kadi M., Tahiri L. (2020). Gastric signet ring cell carcinoma: a comparative analysis of clinicopathologic features. Cancer Control.

[2] Weimann E.T., Botero E.B., Mendes C., Santos M.A.S.D., Stelini R.F., Zelenika C.R.T. (2016). Cutaneous metastasis as the first manifestation of occult malignant breast neoplasia. An Bras Dermatol.

[3] Benesch M.G.K., Mathieson A. (2020). Epidemiology of signet ring cell adenocarcinomas. Cancers (Basel).

[4] Ahn S.J., Oh S.H., Chang S.E. (2007). Cutaneous metastasis of gastric signet ring cell carcinoma masquerading as allergic contact dermatitis. J Eur Acad Dermatol Venereol.

[5] Cokgezer S., Samanci N.S., Bektas M., Kepil N., Demirelli F.H. (2020). Cutaneous metastasis of signet cell gastric carcinoma. Indian J Dermatol.

[6] Acikalin M.F., Vardareli E., Tel N., Saricam T., Urer S. (2005). Erysipelas-like cutaneous metastasis from gastric signet ring cell carcinoma. J Eur Acad Dermatol Venereol.

[7] Bellizzi A.M. (2020). An algorithmic immunohistochemical approach to define tumor type and assign site of origin. Adv Anat Pathol.

[8] Shu Y., Zhang W., Hou Q. (2018). Prognostic significance of frequent CLDN18-ARHGAP26/6 fusion in gastric signet-ring cell cancer. Nat Commun.

[9] Rais G., Bourhafour M., Nafidi F.Z., Rais F. (2022). A huge sister Mary Joseph's nodule from signet ring cell gastric carcinoma showing good response to FOLFOX-based chemotherapy regimen. J Med Cases.

[10] Gorwitz G.L., DeRemer D.L. (2025). Zolbetuximab-clzb: targeting claudin 18.2 in advanced gastric and gastroesophageal adenocarcinoma. Ann Pharmacother.

